# Happiness and associated factors amongst pregnant women in the United Arab Emirates: The Mutaba’ah Study

**DOI:** 10.1371/journal.pone.0268214

**Published:** 2023-01-25

**Authors:** Nasloon Ali, Iffat Elbarazi, Fatima Al-Maskari, Tom Loney, Luai A. Ahmed

**Affiliations:** 1 Institute of Public Health, College of Medicine and Health Sciences, United Arab Emirates University, Al Ain, United Arab Emirates; 2 Zayed Center for Health Sciences, United Arab Emirates University, Al Ain, United Arab Emirates; 3 College of Medicine, Mohammed Bin Rashid University of Medicine and Health Sciences, Dubai, United Arab Emirates; University of Maiduguri College of Medical Sciences, NIGERIA

## Abstract

**Objective:**

Prenatal happiness and life satisfaction research are often over-shadowed by other pregnancy and birth outcomes. This analysis investigated the level of, and factors associated with happiness amongst pregnant women in the United Arab Emirates.

**Methods:**

Baseline cross-sectional data was analyzed from the Mutaba’ah Study, a large population-based prospective cohort study in the UAE. This analysis included all expectant mothers who completed the baseline self-administered questionnaire about sociodemographic and pregnancy-related information between May 2017 and July 2021. Happiness was assessed on a 10-point scale (1 = very unhappy; 10 = very happy). Regression models were used to evaluate the association between various factors and happiness.

**Results:**

Overall, 9,350 pregnant women were included, and the majority (60.9%) reported a happiness score of ≥8 (median). Higher levels of social support, planned pregnancies and primi-gravidity were independently associated with higher odds of being happier; adjusted odds ratio (aOR (95% CI): 2.02 (1.71–2.38), 1.34 (1.22–1.47), and 1.41 (1.23–1.60), respectively. Women anxious about childbirth had lower odds of being happier (aOR: 0.58 (0.52–0.64).

**Conclusion:**

Self-reported happiness levels were high among pregnant women in the UAE. Health services enhancing social support and promoting well-being during pregnancy and childbirth may ensure continued happiness during pregnancy in the UAE.

## Introduction

Happiness is a subjective sense of well-being, joy, or contentment that includes both positive affect and life satisfaction [1]. Previous work has shown that there is a two-way relationship between happiness and health [2]. The level of happiness a person experiences can affect inter-personal relationships and decision making and have long-term consequences on significant aspects of health and important life events [2]. Studies worldwide have sought to understand life satisfaction and quality of life regarding important life events such as pregnancy [3–5].

Pregnancy and childbearing are key life events, and some studies have discussed the happiness of the mother, the father, and the couple before, during, and after childbirth [6, 7]. Pregnancy is a potentially stressful period in a woman’s life, and women with unhappy pregnancies have been shown to engage in negative behaviors, such as tobacco and alcohol consumption, that may be harmful to the mother and fetus [8]. Children born to unhappy mothers tend to experience poorer outcomes at birth, such as lower birth weight and prematurity [9]. On the contrary, happier women tend to engage in more positive health behaviors during their pregnancy [e.g., healthy diet, regular physical activity], leading to optimal birth outcomes such as full-term deliveries, normal birth weight, and early breastfeeding initiation [10, 11].

Currently, there is limited extant scientific literature on the health and well-being of women during pregnancy. There has not been a systematic evaluation of pregnant women’s happiness in the United Arab Emirates (UAE). As such, this analysis aimed to investigate the prevalence of, and factors associated with happiness amongst a representative sample of pregnant women in the UAE.

## Materials and methods

### Study design, setting, and participants

This cross-sectional analysis is based on the questionnaire administered at recruitment from the Mutaba’ah Study. The Mutaba’ah (which means to “follow up” in Arabic) Study methods have been described in detail elsewhere [12]. In brief, the Mutaba’ah Study is a prospective mother and child health cohort that aims to systematically recruit 10,000 pregnant women during their antenatal care visits at the three major hospitals in the city of Al Ain, UAE. All pregnant women from the Emirati population who are at least 18 years old, residents in Al Ain, ideally in their first trimester, and their newborns are eligible to participate in the Mutaba’ah Study. All women recruited in the study provide written consent for their and their children’s participation in the study. The current cross-sectional analysis included data from the baseline self-administered questionnaire administered during the first point of contact with participants who were recruited between May 2017 and February 2021. Ethical approval for the overall cohort study was obtained from the UAE University Human Research Ethics Committee (previously known as Al Ain Medical District Human Research Ethics Committee) (ERH-2017-5512), Al Ain Hospital Research Ethics Committee (AAHEC-03-17-058), and Tawam Human Research Ethics Committee (T-HREC–494).

### Variables

The baseline questionnaire which is administered during the recruitment of the women includes questions on various sociodemographic and pregnancy-related characteristics. The questionnaire includes 67 questions and can be found in the supplementary document. The questions created for the Mutaba’ah study were based on validated questionnaires from other birth cohorts such as the Born in Bradford Study, Norwegian Mother, Father and Child Cohort Study (MoBa), Avon Longitudinal Study of Parents and Children and the Danish National Birth Cohort. They were curated to fit the local population characteristics and cultural sensitivity. The questionnaire was also translated into Arabic from English and then back translated into English to ensure clarity. More on the study and questionnaire can be found in the published protocol [12].

This analysis used questions on maternal age, educational attainment, employment status, perceived social support, the participant’s pregnancy planning status, childbirth anxiety, gestational age, number of pregnancies (gravidity), and happiness. For education, women were asked “What is your highest qualification?”, and those who responded as “Illiterate”, “Never Attended School”, “Primary” or “Secondary” were labeled as “High school and below”, while those who had responded “Vocational or Diploma”, “Bachelors”, “Masters” or “Doctorate” were labeled as “Diploma and above”. Women were labeled as “Employed” if they responded to the employment question “What is your type of work?” as “Employed” or “Self-employed”, and as “Unemployed” if they responded as “Student”, “Housewife”, “Retired” or “Seeking employment”. Women were considered to be primigravid if they reported “Yes” to the question “Is this your first pregnancy?” and were considered gravid if they reported “No”. Social support was assessed using the question: “Do you feel that you have enough people in your life to count on when you need anything?”. Responses were coded as “Yes” if the respondent answered “Yes, enough” and “Yes, definitely enough” and was labeled as a “No” if the respondent answered “No, not much” or “No, not at all”. Women were also asked to report their anxiety towards childbirth and were labeled as a “Yes” if they answered “Yes, quite a lot” or “Yes, sometimes” and “No” if they had answered “No, not at all” or “No, not much”. Happiness was determined using the question: “On a scale of one to ten, with zero being very unhappy and ten being very happy, choose a number on the scale that represents how happy you have been during the last month?”. The variable was then collapsed into two categories–“less happy” and “more happy” with the distinction taking place at the happiness scale’s median score.

### Statistical analyses

Descriptive statistics were performed to explore the distribution of the current analysis’ sample characteristics. Continuous variables were presented as means with standard deviations (SD) or medians with interquartile ranges where appropriate, whilst categorical variables were presented as counts (percentages). Continuous variables with a normal distribution were compared using the Student’s t-test whilst categorical data was compared using the Pearson Chi-square test. Univariate and multivariate logistic regression models were used to quantify the association between different sociodemographic and pregnancy-related characteristics and happiness. Crude (OR) and adjusted odds ratios (aOR) with 95% confidence intervals (CI) were calculated. Missing data was imputed using multiple imputations and was included in additional multivariate regression models. Sensitivity analysis was conducted using happiness (i) with a cut-off at mid-point (score of 5); (ii) as tertiles (three groups); and (iii) as a continuous variable. Ordinal and linear regressions were used when happiness was treated as tertiles and continuous variables, respectively. Statistical analyses were performed using Stata 16.1 (Stata Corp, College Station, TX). A p-value of ≤0.05 defined statistical significance.

## Results

There were 10,565 women recruited during the period of May 2017 to July 2021. Pregnant women had a mean (±SD) age of 31.0±6.0 years. The majority (78.5%) of the current cohort were parous (had one or more children prior to the index pregnancy), and the mean (± SD) parity was 2±2 children. Overall, 9,350 (88.5%) women reported their happiness levels. There were no significant differences in terms of maternal age, gestational age, or the number of children between the women who did and did not respond to the happiness question.

**Fig 1:**
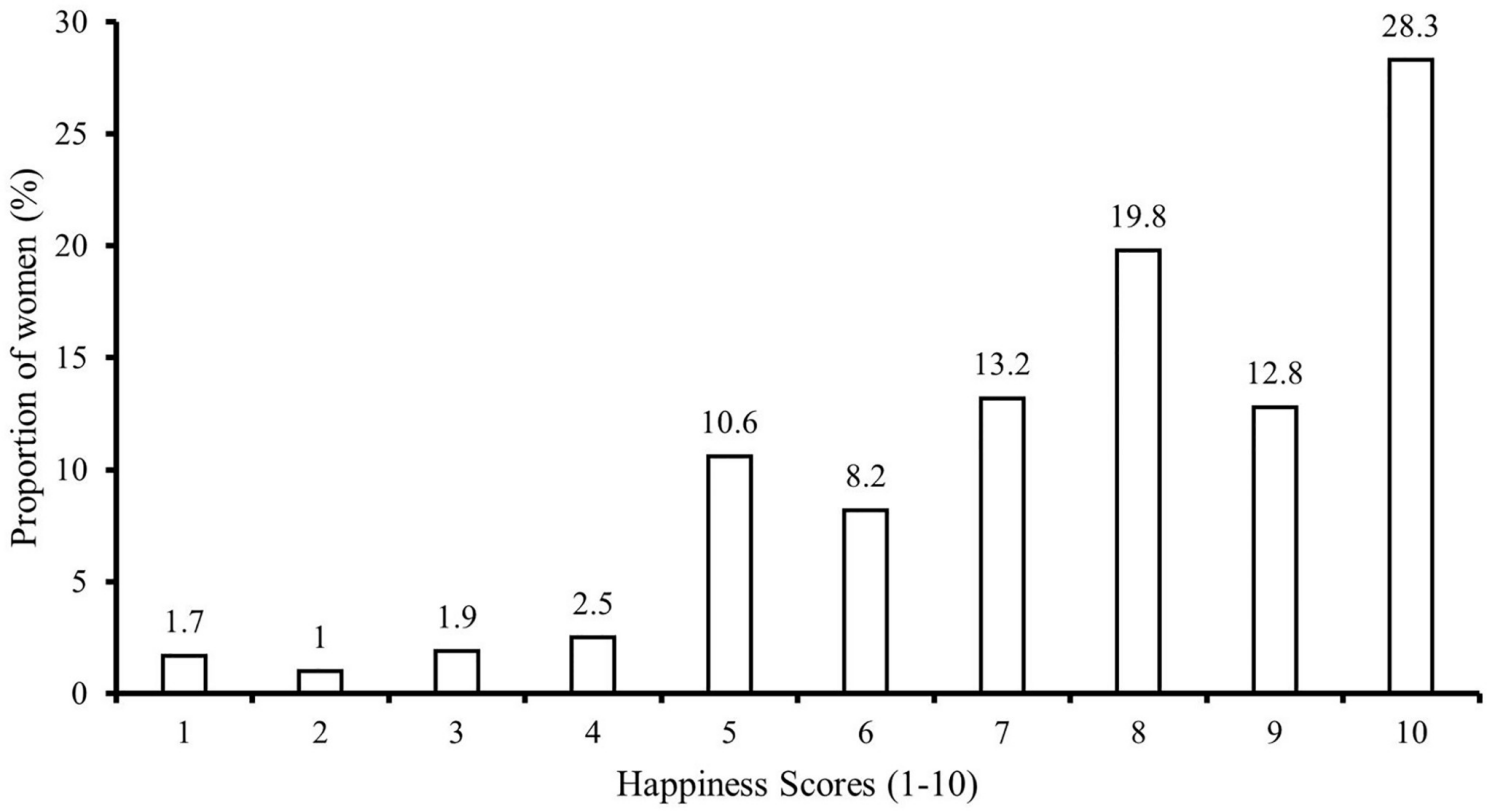
Distribution of happiness scores amongst 9,350 pregnant women in Al Ain, UAE. The Mutaba’ah Study.

The mean (± SD) happiness score was 7.7 ± 2.2 and the median score was 8 (IQR: 6–10) for the included pregnant women. Majority (60.9%) of the women reported their happiness as ≥8 whilst more than a quarter (28.4%) perceived themselves to be “very happy” (maximum score of 10) (Fig 1).

Table 1 shows the characteristics of pregnant women by happiness score level. Women with a self-reported happiness score of 8 or more “more happy” were primigravida at the current pregnancy, were more likely to have higher education, more social support, planned pregnancy, and less worry about childbirth compared to “less happy” women, all p-values ≤0.05. There were no significant differences in maternal age, gestational age, or employment status between the two groups (Table 1).

**Table 1:** Characteristics of 9,350 pregnant women according to self-reported happiness score in Al Ain, UAE. The Mutaba’ah Study.

	Less Happy	More Happy	*P-value* *
	*(Score 1–7)*	*(Score 8–10)*	
N (%)	3,658 (39.1%)	5,692 (60.9%)	
Age (years)	31.3±6.0	30.9±6.0	*0*.*002*
Gestational Age (months)	5.8±2.4	5.0±2.3	*0*.*163*
Gravidity			*<0*.*001*
Primigravid (first pregnancy)	646 (17.9%)	1,331 (23.6%)	
Multigravida	2,972 (82.1%)	4,309 (76.4%)	
*Missing*	*40*	*52*	
Employment			*0*.*259*
Employed	1,223 (33.7%)	1.846 (32.6%)	
Unemployed	2,409 (66.3%)	3,826 (67.4%)	
*Missing*	*26*	*20*	
Education			*0*.*002*
High school and below	2,171 (59.7%)	3,203 (56.5%)	
Diploma and above	1,465 (40.3%)	2.471 (43.5%)	
*Missing*	*22*	*18*	
Perceived Social Support			*<0*.*001*
Yes	3,164 (87.0%)	5,311 (93.6%)	
No	472 (13.0%)	361 (6.4%)	
*Missing*	*22*	*20*	
Planned Pregnancy			*<0*.*001*
Yes	1,823 (50.7%)	3,298 (58.9%)	
No	1,774 (49.3%)	2,303 (41.1%)	
*Missing*	*61*	*91*	
Worrying about Birth			*<0*.*001*
Yes	2,675 (73.7%)	3,480 (61.3%)	
No	957 (26.3%)	2,198 (38.7%)	
*Missing*	*26*	*14*	

Happiness cut-off level is based on the median value.

Data are presented as means ± standard deviation or n (%) [unless otherwise specified].

*P values were found using the chi-square or student’s t-test.

**Fig 2:**
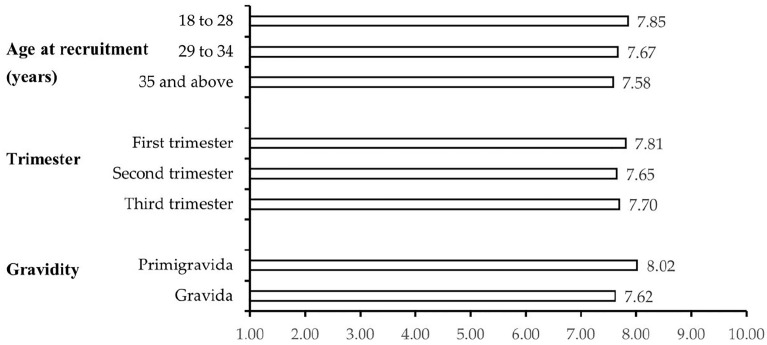
Distribution of median happiness scores by demographic groups amongst pregnant women in Al Ain, UAE. The Mutaba’ah Study.

Median happiness scores between age groups, trimesters, and gravidity are illustrated in Fig 2. Despite trends existing between gravidity and maternal age, the figure shows that median scores of happiness vary only slightly and all between 7 and 8.

The crude associations between the sociodemographic and pregnancy-related characteristics and level of happiness (“less happy” and “more happy”) are presented in Table 2. Women who perceived greater social support (OR: 2.19, 95% CI: 1.90–2.53), and those who were more educated (OR: 1.14, 95% CI: 1.05–1.24), primigravid (OR: 1.42, 95% CI 1.27–1.58), or had planned their pregnancy (OR: 1.39, 95% CI: 1.28–1.52) were more likely to report higher happiness scores. Women who were anxious about their delivery (OR: 0.57, 95% CI: 0.52–0.62) reported lower happiness levels. Maternal age and employment were not significantly associated with happiness.

**Table 2:** Crude and adjusted associations between sociodemographic and pregnancy-related factors and self-reported levels of happiness in pregnant women in Al Ain, UAE. The Mutaba’ah Study.

	Crude Odds Ratio (95% CI)	Adjusted Odds Ratio (95% CI) ^a^	Adjusted Odds Ratio (95% CI) via MI ^b^
Employment	0.95 (0.87–1.04)	0.96 (0.86–1.07)	0.94 (0.85–1.03)
Education*	1.14 (1.05–1.24)	1.10 (0.99–1.21)	1.10 (1.00–1.20)
Perceived Social Support**	2.19 (1.90–2.53)	2.02 (1.71–2.38)	2.04 (1.77–2.37)
Planned Pregnancy**	1.39 (1.28–1.52)	1.34 (1.22–1.47)	1.36 (1.24–1.48)
Worry about Birth**	0.57 (0.52–0.62)	0.58 (0.52–0.64)	0.56 (0.51–0.61)
Primi-gravida**	1.42 (1.28–1.58)	1.41 (1.23–1.60)	1.43 (1.27–1.61)

Adjusted models included all covariates in addition to age and gravidity. MI: multiple imputation

^a^ included 7,647 women with non-missing values of all covariates

^b^ included 9,350 women (imputed values for missing data)

*p<0.05,

**p<0.001

In Table 2, the multivariate analyses showed that pregnant women who perceived more social support were twice more likely to report being happier compared to those who did not perceive more social support (aOR: 2.02 95% CI 1.71–2.38). Pregnant women who reported planned pregnancies were 34% more likely to have higher happiness levels than women who did not report planned pregnancies (aOR: 1.34, 95% CI 1.22–1.47). Women were primi-gravida or having their first child in the index pregnancy were also about 41% more likely to report higher levels of happiness (aOR: 1.41, 95%CI 1.23–1.60). Women who were worried about childbirth were 42% less likely to report high levels of happiness compared to those who did not report childbirth anxiety (aOR: 0.58, 95% CI 0.52–0.64). Multivariate models with imputed data show similar estimates of the associations with happiness (Table 2). Sensitivity analysis with happiness defined from the midpoint of 5, as tertiles, or as a continuous scale did not show variations from the findings above (S1 Table). However, it is worthwhile to mention that education was independently associated with happiness when the outcome variable was treated as a dichotomous variable with a cut-off score of 5 (aOR: 1.38, 95% CI: 1.20–1.58)

## Discussion

Self-reported happiness levels were high in this population of pregnant women in the UAE. Social support, pregnancy planning, education, and anxiety about childbirth were significantly associated with self-reported happiness.

Overall, most of the participants in this analysis (61%) reported a high level of happiness (score ≥8 out of 10) while more than a quarter perceived themselves to be “very happy” (i.e., maximum score of 10). According to the World Happiness Report 2020 [13], the UAE is currently ranked 21st for happiness with respect to all residents of the country. Inclusion of happiness in the policies, programs, and services, and the promotion of positivity and happiness as a lifestyle in the community are among the main areas covered by the UAE National Program for Happiness and Positivity. Satisfaction with healthcare availability is an important reason for happiness in the UAE, and this could have been one of the factors contributing to the high levels of happiness in this sample of pregnant women.

The social environment is a crucial determinant of happiness [13]. Previous research has shown that people can count on their family and network of friends reported higher levels of happiness [14–16]. We found similar results in this analysis as pregnant women with greater social support were twice as likely to be in the “more happy” group. Expectant mothers can experience a wide range of physical, mental, and emotional stressors that can lead to unfavorable health outcomes [17–19]. Adequate social support can help pregnant women diminish this anxiety. Positive associations between perceived support and happiness levels could be because happier people have better social skills [20] and may have enhanced joy and quality of life arising from these satisfying social relations [21].

Pregnancy intention has been shown to affect pregnant women’s happiness levels in diverse nationalities [8]. A planned pregnancy has been shown to be directly related to prenatal happiness [22]. Women with planned pregnancies are likely happier due to being more conducive to planning their pregnancy and impending childbirth [23]. It is noteworthy that the development of life satisfaction is similar in planned and unplanned pregnancies when the trajectory is studied as women achieve similar satisfaction gains due to motherhood [24]. However, our study findings show increased odds for women with planned pregnancies to be in the happier category. Future research may want to explore the relationships between unplanned pregnancies and happiness and life satisfaction in more detail using sub-categories related to desire and pregnancy timing.

Moreover, like previous studies [25, 26], fear of childbirth was less frequent amongst happier pregnant women in this analysis Regardless of its root cause, anxious behavior has been shown to influence individual happiness negatively [27]. Earlier work has reported that the perceived ability to cope with forthcoming childbirth is sometimes frightening and can lead to lower levels of happiness, especially among nulliparous women [28, 29]. Research has shown that psychoeducational programs during pregnancy can facilitate expectant mothers to develop the appropriate coping mechanisms to reduce anxiety and negative emotions related to childbirth [30]

Although some previous studies have shown that happier pregnant women tend to be older, unemployed, or have a higher household income [31–35], this analysis could not confirm these characteristics. Employment or income status might show no associations with happiness level as all participants in this study have similar levels of access to high-quality healthcare. Globally, happiness levels have shown to differ by parity or gravidity but are modified by individual factors [36]. Although the first child or pregnancy is positively related to happiness, fluctuations in well-being as the number of children increase in the household differ based on contextual factors such as good health and income which were not explicitly included in this analysis. Noting the median levels in Fig 2 stratified by gravidity, happiness seems to peak at the first pregnancy in this analysis. In other research, it has been stipulated that happiness levels elevate in the year of the birth and return to pre-birth levels after and plateaus until the child reaches adult ages [7]. Furthermore, whilst education was not significantly associated with happiness [defined based on the median score], it was independently associated with happiness treated as a dichotomized variable from the midpoint score of 5. Indeed, previous studies have also shown that education affects happiness in both the general populations of all ages and nationalities [4], and pregnancy-related happiness [36]. Pregnant women who are more educated may be more knowledgeable about their pregnancy and may be more likely to achieve a stable state of well-being and even enjoy their pregnancy.

The happiness and subjective well-being literature consistently mention that global judgments on happiness do not consider average mood or level of satisfaction. Therefore, it is also necessary to employ qualitative research methods to explore the meaning, value, and interpretation of happiness as a concept among pregnant women in the UAE. Moreover, further exploration of the associations between happiness and various maternal, infant, and child health outcomes is warranted.

### Strengths and limitations

To our knowledge, this is the largest analysis to investigate the factors associated with happiness amongst pregnant women in the region. The cross-sectional analysis from the Mutaba’ah Study included a large representative sample of the population from both public and private hospitals to prevent selection bias. This allows for the generalization of the results to the population of pregnant women in Al Ain. The recall time for happiness was limited to one month, which ensured that it reflected the period during pregnancy. This study included a focused subset of factors that might influence a woman’s happiness during her pregnancy based on previously published data on prenatal happiness [15, 17]. Similar to other cross-sectional analyses, the reported associations do not indicate causality. Using a single item to assess a complex perceived sense of well-being may be viewed as simplistic and a limitation of the study. However, measuring happiness by a single unambiguous item could also considered as a strength. The available lengthy psychometrically validated self-report instruments would not have been suitable for the data collection process used in this study design [i.e., a long baseline questionnaire collecting a wide range of sociodemographic and pregnancy-related variables]. Moreover, this approach is aligned with other cohort studies collecting a large and varied dataset [7] and is found to be reliable and valid as it is significantly and positively correlated with other validated instruments [36]. Nevertheless, as the study continues to expand and recruitment has been deemed feasible in the Mutaba’ah study, the research personnel will endeavor to include validated instruments such as the Oxford happiness questionnaire to assess well-being.

## Conclusions

The happiness levels of pregnant women currently enrolled in the Mutaba’ah Study were found to be high. Health services focusing on enhancing social support and promoting a positive well-being about pregnancy and childbirth may ensure continued happiness during pregnancy and beyond among women in the UAE.

## Supporting information

S1 Table Characteristics of participants when happiness is a dichotomous variable with cut-off at 5. (DOCX)

S2 Table Crude and adjusted associations between sociodemographic and pregnancy-related factors and self-reported levels of happiness (as a dichotomous variable) in pregnant women in Al Ain, UAE. The Mutaba’ah Study.
(DOCX)

S3 Table Characteristics of participants when happiness is an ordinal variable with three categories separated as tertiles. (DOCX)

S4 Table Crude and adjusted associations between sociodemographic and pregnancy-related factors and self-reported levels of happiness (tertiles) in pregnant women in Al Ain, UAE. The Mutaba’ah Study.
(DOCX)

S5 Table Crude and adjusted associations between sociodemographic and pregnancy-related factors and self-reported levels of happiness (as a continuous table) in pregnant women in Al Ain, UAE. The Mutaba’ah Study.
(DOCX)
