# Supplementary material for: Happiness and associated factors amongst pregnant women in the United Arab Emirates: The Mutaba’ah Study
Source: PLoS One. 2023 Jan 25;18(1):e0268214. doi: 10.1371/journal.pone.0268214 (PMC9876351; doi:10.1371/journal.pone.0268214)
Supplement: S1 Table — (DOCX) [file pone.0268214.s001.docx]

S1 Table: Characteristics of participants when happiness is a dichotomous variable with cut-off at 5

| Factor of interest | More Happy (6-10)  (n=8,901) | Less happy (1-5)  (n=1,661) | p-value |
| --- | --- | --- | --- |
| Age | 30.9±6.0 | 31.6±6.1 | <0.001 |
| Gestational age | 5.80±2.40 | 5.79±2.31 | 0.858 |
| Gravidity*  Primigravid  Multigravida |  |  | <0.0001 |
|  | 1,875 (22.6) | 260 (15.8) |  |
|  | 6,427 (77.4) | 1,383 (84.2) |  |
| Planned Pregnancy*  Yes  No |  |  | <0.001 |
|  | 4,667 (57.3) | 754 (46.0) |  |
|  | 3,482 (42.7) | 885 (54.0) |  |
| Worrying about birth*  Yes  No |  |  | <0.001 |
|  | 5,060 (63.9)  2,858 (36.1) | 1,255 (76.3)  390 (23.7) |  |
| Social Support *  Yes  No |  |  | <0.001 |
|  | 7,356 (92.9)  562 (7.1) | 1,350 (82.1)  294 (17.9) |  |
| Education*  High school and below  Diploma and above |  |  | <0.001 |
|  | 4,525 (56.4)  3,500 (43.6) | 1,084 (65.6)  568 (34.4) |  |
| Employment*  Employed  Not employed |  |  | 0.357 |
|  | 2,653 (33.1) | 527 (31.9) |  |
|  | 5,360 (66.9) | 1,123 (68.1) |  |

*Note: * denotes missing values excluded due to non-response*
