# Supplementary material for: Happiness and associated factors amongst pregnant women in the United Arab Emirates: The Mutaba’ah Study
Source: PLoS One. 2023 Jan 25;18(1):e0268214. doi: 10.1371/journal.pone.0268214 (PMC9876351; doi:10.1371/journal.pone.0268214)
Supplement: S2 Table — The Mutaba’ah Study. (DOCX) [file pone.0268214.s002.docx]

**S2 Table: Crude and adjusted associations between sociodemographic and pregnancy-related factors and self-reported levels of happiness (as a dichotomous variable) in pregnant women in Al Ain, UAE. The Mutaba’ah Study**

|  | **Crude Odds Ratio (95% CI)** | **Adjusted Odds Ratio (95% CI)** *^a^* | **Adjusted Odds Ratio (95% CI) via MI** *^b^* |
| --- | --- | --- | --- |
| Employment | 1.05 (0.94-1.18) | 0.97 (0.84-1.12) | 0.94 (0.82-1.08) |
| Education* | 1.48 (1.32-1.65) | 1.39 (1.21-1.59) | 1.38 (1.20-1.58) |
| Perceived Social Support** | 2.85 (2.45-3.32) | 2.58 (2.15-3.09) | 2.53 (2.11-3.02) |
| Planned Pregnancy** | 1.57 (1.41-1.75) | 1.47 (1.29-1.66) | 1.43 (1.26-1.62) |
| Worry about Birth** | 0.55 (0.49-0.62) | 0.55 (0.48-0.63) | 0.56 (0.49-0.65) |
| Primi-gravida** | 1.55 (1.35-1.79) | 1.36 (1.11-1.67) | 1.36 (1.12-1.66) |

Adjusted models included all covariates in addition to age and gravidity.

MI: multiple imputation
^a^ included 7,647 women with non-missing values of all covariates
^b^ included 9,350 women (imputed values for missing data*)*

*p<0.05, **p<0.001
