# Supplementary material for: Happiness and associated factors amongst pregnant women in the United Arab Emirates: The Mutaba’ah Study
Source: PLoS One. 2023 Jan 25;18(1):e0268214. doi: 10.1371/journal.pone.0268214 (PMC9876351; doi:10.1371/journal.pone.0268214)
Supplement: S3 Table — (DOCX) [file pone.0268214.s003.docx]

**S3 Table: Characteristics of participants when happiness is an ordinal variable with three categories separated as tertiles**

| **Factor of interest** | **Tertile 1 (Scores 1-7)**  **N= 3,658** | **Tertile 2 (Scores 8 & 9)**  **N = 3,043** | **Tertile (10)**  **N= 2,649** | **p-value** |
| --- | --- | --- | --- | --- |
| Age | 31.2±6.0 | 31.0±6.0 | 30.7±6.0 | 0.976 |
| Gestational age | 5.86±2.3 | 5.96±2.4 | 5.60±2.5 | <0.001 |
| Gravidity*  Primigravid  Multigravida |  |  |  | <0.001 |
|  | 646 (17.9) | 665 (22.1) | 666 (25.4) |  |
|  | 2972 (82.1) | 2351 (77.9) | 1958 (74.6) |  |
| Planned Pregnancy*  Yes  No |  |  |  | <0.001 |
|  | 1,823 (50.7) | 1777 (59.2) | 1521 (58.5) |  |
|  | 1774 (49.3) | 1225 (40.8) | 1078 (41.5) |  |
| Worrying about birth*  Yes  No |  |  |  | <0.001 |
|  | 2675 (75.7) | 1960 (64.6) | 1520 (57.5) |  |
|  | 957 (26.3) | 1075 (35.4) | 1123 (42.5) |  |
| Social Support*  Yes  No |  |  |  | <0.001 |
|  | 3164 (87.0) | 2830 (93.3) | 2481 (94.0) |  |
|  | 472 (13.0) | 203 (6.7) | 158 (6.0) |  |
| Education*  High school and below  Diploma and above |  |  |  | <0.001 |
|  | 1465 (40.3) | 1473 (48.5) | 998 (37.8) |  |
|  | 2171 (59.7) | 1562 (51.5) | 1641 (62.2) |  |
| Employment*  Employed  Not employed |  |  |  | 0.001 |
|  | 1223 (33.7) | 1051 (34.7) | 795 (30.1) |  |
|  | 2409 (66.3) | 1981 (65.3) | 1845 (69.9) |  |

*Note: * denotes missing values excluded due to non-response*
