# Supplementary material for: Happiness and associated factors amongst pregnant women in the United Arab Emirates: The Mutaba’ah Study
Source: PLoS One. 2023 Jan 25;18(1):e0268214. doi: 10.1371/journal.pone.0268214 (PMC9876351; doi:10.1371/journal.pone.0268214)
Supplement: S4 Table — The Mutaba’ah Study. (DOCX) [file pone.0268214.s004.docx]

**S4 Table: Crude and adjusted associations between sociodemographic and pregnancy-related factors and self-reported levels of happiness (tertiles) in pregnant women in Al Ain, UAE. The Mutaba’ah Study**

|  | **Crude Odds Ratio (95% CI)** | **Adjusted Odds Ratio (95% CI)** *^a^* | **Adjusted Odds Ratio (95% CI) via MI** *^b^* |
| --- | --- | --- | --- |
| Employment | 0.90 (0.83-0.97) | 0.94 (0.85-1.04) | 0.92 (0.83-1.01) |
| Education* | 0.97 (0.90-1.05) | 0.89 (0.81-0.98) | 0.90 (0.82-1.00) |
| Perceived Social Support** | 2.08 (1.81-2.39) | 1.92 (1.63-2.26) | 1.91 (1.62-2.24) |
| Planned Pregnancy** | 1.20 (1.20-1.40) | 1.26 (1.15-1.37) | 1.26 (1.16-1.38) |
| Worry about Birth** | 0.58 (0.53-0.63) | 0.58 (0.53-0.63) | 0.59 (0.53-0.64) |
| Primi-gravida** | 1.40 (1.28-1.54) | 1.32 (1.15-1.51) | 1.31 (1.15-1.50) |

Adjusted models included all covariates in addition to age and gravidity. Reference group is Tertile 1

MI: multiple imputation
^a^ included 7,647 women with non-missing values of all covariates
^b^ included 9,350 women (imputed values for missing data*)*

*p<0.05, **p<0.001
