# Supplementary material for: Happiness and associated factors amongst pregnant women in the United Arab Emirates: The Mutaba’ah Study
Source: PLoS One. 2023 Jan 25;18(1):e0268214. doi: 10.1371/journal.pone.0268214 (PMC9876351; doi:10.1371/journal.pone.0268214)
Supplement: S5 Table — The Mutaba’ah Study. (DOCX) [file pone.0268214.s005.docx]

**S5 Table: Crude and adjusted associations between sociodemographic and pregnancy-related factors and self-reported levels of happiness (as a continuous table) in pregnant women in Al Ain, UAE. The Mutaba’ah Study**

|  | **Crude Odds Ratio (95% CI)** | **Adjusted Odds Ratio (95% CI)** *^a^* | **Adjusted Odds Ratio (95% CI) via MI** *^b^* |
| --- | --- | --- | --- |
| Employment | 0.94 (0.86-1.04) | 0.94 (0.84-1.05) | 0.91 (0.81-1.02) |
| Education* | 1.17 (1.07-1.28) | 1.07 (0.97-1.20) | 1.09 (0.98-1.21) |
| Perceived Social Support** | 3.03 (2.60-3.52) | 2.72 (2.29-3.23) | 2.70 (2.27-3.21) |
| Planned Pregnancy** | 1.43 (1.31-1.57) | 1.31 (1.18-1.44) | 1.32 (1.19-1.45) |
| Worry about Birth** | 0.55 (0.50-0.61) | 0.56 (0.51-0.63) | 0.57 (0.51-0.63) |
| Primi-gravida** | 1.50 (1.34-1.66) | 1.31 (1.13-1.53) | 1.31 (1.12-1.52) |

Adjusted models included all covariates in addition to age and gravidity.

MI: multiple imputation
^a^ included 7,647 women with non-missing values of all covariates
^b^ included 9,350 women (imputed values for missing data*)*

*p<0.05, **p<0.001
